# Determination of X-ray flux using silicon pin diodes

**DOI:** 10.1107/S0909049508040429

**Published:** 2009-02-25

**Authors:** Robin L. Owen, James M. Holton, Clemens Schulze-Briese, Elspeth F. Garman

**Affiliations:** a Swiss Light Source, Paul Scherrer Institute, CH-5232 Villigen PSI, Switzerland; b Department of Biochemistry and Biophysics, University of California, San Francisco, CA 94158-2330, USA; c Advanced Light Source, Lawrence Berkeley National Laboratory, Berkeley, CA 94720, USA; d Laboratory of Molecular Biophysics, Department of Biochemistry, University of Oxford, South Parks Road, Oxford OX1 3QU, UK

**Keywords:** macromolecular crystallography, flux determination, silicon pin diode, absorbed dose

## Abstract

Accurate measurement of photon flux from an X-ray source, a parameter required to calculate the dose absorbed by the sample, is not yet routinely available at macromolecular crystallography beamlines. The development of a model for determining the photon flux incident on pin diodes is described here, and has been tested on the macromolecular crystallography beamlines at both the Swiss Light Source, Villigen, Switzerland, and the Advanced Light Source, Berkeley, USA, at energies between 4 and 18 keV. These experiments have shown that a simple model based on energy deposition in silicon is sufficient for determining the flux incident on high-quality silicon pin diodes. The derivation and validation of this model is presented, and a web-based tool for the use of the macromolecular crystallography and wider synchrotron community is introduced.

## Abstract 2

Accurate measurement of photon flux from an X-ray source is a parameter required to calculate the dose absorbed by a sample. The development of a model for determining the photon flux incident on pin diodes, and experiments to test this model, are described for incident energies between 4 and 18 keV used in macromolecular crystallography.

## 1. Introduction

With the recent resurgence of interest in radiation damage progression and avoidance in crystals used for macromolecular crystallography (MX), knowledge of the dose absorbed during an experiment is becoming increasingly important. Dose is the amount of energy per unit mass (J kg^−1^ or Gy) deposited in the sample and is proving to be the fundamental coordinate of radiation damage progression (Ravelli & Garman, 2006 ▶). This is because at cryotemperatures of around 100 K the damage appears to depend on the accumulated energy absorbed by the sample, regardless of the time taken to deposit it [*i.e.* is largely independent of dose rate (Owen *et al.*, 2006 ▶; Leiros *et al.*, 2006 ▶; Sliz *et al.*, 2003 ▶)]. The upper dose limit that can be tolerated by a macromolecular crystal held at 100 K before half of its diffraction intensity is lost has been predicted from electron microscopy observations [20 MGy (Henderson, 1990 ▶)] and measured for MX [43 MGy (Owen *et al.*, 2006 ▶)]. However, data collection in MX is typically formulated in units of time, so optimal planning of experiments, as well as measuring and comparing damage rates, requires that the relationship between dose and time be established for both the source and the sample in use.

The dose absorbed by a crystal can be calculated from the physics of the interaction between X-rays and atoms, but a prerequisite to this calculation is knowledge of the size, shape and intensity (flux: photons s^−1^) of the incident X-ray beam (Murray *et al.*, 2004 ▶; Paithankar *et al.*, 2009 ▶). These parameters are not yet routinely measured at all synchrotron MX beamlines, and this paper will focus on how accurate determination of X-ray photon flux can be achieved.

### 1.1. Counting devices

The concept of measuring photon flux is deceptively simple: some number of X-ray photons emerge from the collimator every second. Counting each photon is arguably the most accurate method of measuring the intensity of the beam, since the uncertainty of a photon count is limited only by the fundamentally random nature of photon arrivals. Photon flux obeys Poisson statistics, for which the error in counting *N* photons in a given amount of time is *N* ^1/2^. For example, if 10000 photons are counted in 1s, it can be concluded that the beam flux is 10^4^ photons s^−1^ with a statistical error of ±100 photons s^−1^ or 1%. Counting for a longer time will accumulate more photons and thus improve the signal-to-noise ratio and statistical accuracy further.

Unfortunately, there are no X-ray photon counting devices appropriate for direct measurement of the high fluxes produced by modern X-ray sources. While a detailed discussion of detector technologies is beyond the scope of this work, it is sufficient to note here that the fastest modern counting detectors are still several orders of magnitude too slow to count a 100 × 100 µm beam of 10^12^ photons s^−1^. This is because counting anything requires that the objects to be counted be identified individually, which becomes increasingly difficult as the time interval between photon arrivals decreases. For a given counting device, the minimum time between two photon hits that still register as two separate events is called the dead-time because it reflects the period of time for which the detector electronics are processing one photon and thus cannot yet detect the arrival of another. As detector technology improves, this dead-time is being reduced, but it can never be zero, so some fraction of the photons arriving at any counting detector will invariably be missed.

The observed count rate will therefore always be less than the true count rate, but for simple paralyzable detectors such as the scintillation counter described below the ratio between these rates can be derived from the Poisson distribution. The latter can be used to correct an observed count rate, provided that the true count rate is less than one photon per dead-time interval (Lucke, 1976 ▶). Implementation of this dead-time correction enables counting detectors to have a linear response over a large count rate range, from as low as a few photons s^−1^ up to almost 10^7^ photons s^−1^ if the device has a dead-time of 100 ns. However, the counting of 10^12^ photons s^−1^ requires a detector dead-time of less than 1 ps, well beyond the limit of current detectors. It should also be noted that counting detectors themselves suffer from radiation damage, and the response of such a detector may therefore vary over time.

### 1.2. Attenuators

A solution to this problem would be to place an absorber in the beam to reduce the flux to a manageable value. However, the accuracy of high attenuation factors is very sensitive to the thickness and composition of the absorber. For example, a 1000-fold attenuator is only 5% thinner than a 1400-fold attenuator, making extrapolation to full flux problematic and inaccurate given even small uncertainties in the thickness. In addition, any practical attenuator that absorbs strongly at a given photon energy will not stop a photon of three times that energy effectively, and this results in additional error in using the attenuation method. These high-energy ‘harmonic contamination’ photons usually represent only a small fraction of the beam and are not normally significant in MX experiments. However, a counting detector behind a ∼1000-fold aluminium absorber would experience these photons with almost no attenuation, giving a significant error if a non-energy-discriminating detector is used.

### 1.3. Calorimetry

An alternative method of quantifying X-ray flux would be to measure the sample heating induced by the beam. Given the large fluxes achievable at third-generation sources, one might expect that enough heat would be deposited in a detector to allow accurate measurement. However, the total power in a beam of 10^12^ photons s^−1^ and 1 Å wavelength (12.4 keV) is relatively small at ∼2 mW^1^, roughly equivalent to the power emitted by a hand-held laser pointer, and this is difficult to measure accurately. For example, a 1 mm^3^ diode temperature sensor wrapped with a small heating resistor inside an insulating foam block increased in temperature by no more than 0.05 K when the resistor was dissipating 1 mW, and no change could be detected at 0.1 mW (data not shown), indicating that even 10% accuracy was unattainable with this arrangement. Considerable effort and technical ingenuity was expended measuring the heat deposited by a high X-ray flux (3 × 10^12^ photons s^−1^, FWHM of beam 103 × 84 µm, energy 6.5 keV) into a glass bead which induced temperature increases of only a few degrees (Snell *et al.*, 2007 ▶). The difficulties in carrying out measurements of this type arise because small objects dissipate heat very efficiently, and temperature fluctuations of the order of 0.1 K are difficult to avoid. In practice, accurate X-ray measurements in the sub-mW range require cooling of the calorimeter to liquid-helium temperatures, at which the heat capacities of most materials are extremely low, low enough to outpace dissipation. An example of such a device is the cryogenic electrical substitution radiometer which serves as an absolute reference detector at the Physikalisch–Technische Bundesanstalt beamline at BESSY (Gerlach *et al.*, 2007 ▶). Such devices are difficult to build, calibrate and maintain, and although they make good reference detectors they are not suitable for routine flux measurements at an MX beamline.

### 1.4. Ionization chambers

Ionization chambers are frequently used to measure X-ray photon flux both at synchrotron beamlines and in medical dosimetry (see, for example, http://www.npl.co.uk/server.php?show=ConWebDoc.305). A detailed description of the design and use of ion chambers is beyond the scope of this paper, but generally these devices consist of two metal plates on either side of a gas-filled box with a voltage applied across the plates. Voltages of order 100–2000 V cm^−1^ are required to avoid space charge saturation for highly brilliant X-ray beams (Wyckof, 1979 ▶; Nariyama, 2006 ▶). X-ray photons with the wavelengths used for MX interact with matter in one of three ways: photoelectric emission, elastic scattering or Compton scattering. Both photoelectric emission and Compton scattering result in ionization, but photoelectric emission is the dominant process at MX energies and Compton scattering can be neglected; the error introduced by this approximation is <5%. The ions and free electrons generated by ionization cause a current proportional to the energy lost by the photon beam to flow between the plates. For a well designed and correctly biased chamber, the proportionality constant is the average energy required to produce an electron–ion pair in the gas, which is most well established for air at 33.85 ± 0.15 eV (Wyckof, 1979 ▶).

This absolute calibration is a valuable feature of ionization chambers, but a disadvantage of these devices is the size of the chamber and accompanying pair of guard plates which are typically each ∼30 mm in length. In general, it is desirable for flux sensors to be small so that their design impact on the beamline is negligible. For example, it is convenient to avoid disturbing the beamstop and sample for each flux measurement, but this requires that the flux sensor be small enough to be inserted between these two components. It is, however, not a simple task to miniaturize ion chambers as, in devices smaller than the track length of photoelectrons in air [∼3 mm for 12 keV X-rays (Cole, 1969 ▶)], photoelectrons interact directly with the plates and the calibration must be determined empirically (Nariyama, 2004 ▶; Kocsis & Somogyi, 2003 ▶). Increasing the density of the ionizing medium in the chamber decreases the track length and allows the simple electron–ion pair creation model to be recovered. Thus a very small low-voltage ionization chamber that uses a dense solid instead of a gas would be ideal for routine flux measurements at MX beamlines. The silicon photodiode is such a device (Jach & Cowan, 1983 ▶; Krumrey *et al.*, 2006 ▶; Alkire & Rotella, 1997 ▶).

### 1.5. Silicon pin diodes

In an analogous process to that described above for ion chambers, absorption of a photon in a silicon crystal creates separations of charge, which are called electron–hole pairs and require an average energy, ∊, of 3.66 ± 0.03 eV (Alig & Bloom, 1975 ▶; Scholze *et al.*, 2000 ▶) for generation. Physically, electrons and holes are charged electronic excited states of atoms in the crystal lattice, which move under the influence of electric fields, such as that caused by the difference in chemical potential between the p (boron or aluminium doped) and n (phosphorus, arsenic or antimony doped) layers, resulting in the flow of an electric current.

**Figure 1:**
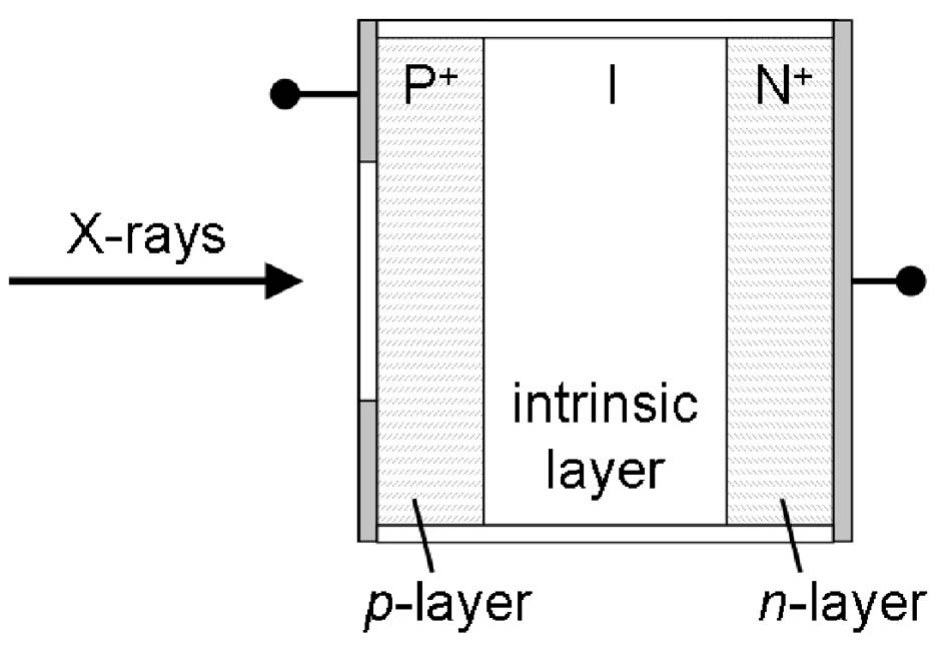
Diagrammatic representation of a pin type diode, showing a typical P^+^–I–N^+^ (pin) layer arrangement.

In order to prevent rapid recombination of the electron–hole pairs, one of two methods is generally used: a reverse bias voltage can be applied to the diode which has the effect of increasing the width of the depletion region between the p and n regions, or a pin diode can be used (Fig. 1 ▶). A pin photodiode has a large intrinsic silicon (i) layer containing no added impurities between the p and n regions, and any carriers formed in this region rapidly cross the junction, resulting in a photocurrent. By calculating the amount of energy deposited in this layer by the photoelectric effect, this photocurrent, *I*, can be related to an X-ray photon flux, ϕ.

Despite the introduction of an i layer, it might be expected that the electrons and holes will be attracted to one another and quickly recombine to generate heat. However, direct recombination is a forbidden process in indirect band-gap semiconductors such as silicon, and it must be assisted by a lattice vibration to conserve momentum. In practice, the fastest way for electrons and holes to recombine is at defect sites in the silicon crystal lattice. Thus, a high-quality silicon pin diode should exhibit little or zero charge carrier recombination.

For MX, flux measurements can most conveniently be made using calibrated pin diodes, and these are now being used routinely at a number of beamlines both by beamline staff and by visiting experimenters, in particular those researching various aspects of radiation damage. Of interest is the pin diode reproducibility and ease of use over the X-ray energy range used for MX, as well as the reliability and accuracy of converting the measured currents into photon flux using a simple model which relates the energy lost by the X-rays *via* the photoelectric effect to the current induced in the diode. A comparison of various diodes of different types and thicknesses carried out both at the Advanced Light Source (ALS, Berkeley, USA) and the Swiss Light Source (SLS, Villigen, Switzerland) is presented here, as well as a model by which flux can most easily be quantified to enable accurate MX dose calculations. A web-based tool to facilitate computation of photon flux from the current induced in pin diodes is presented.

## 2. Materials and methods

In this section the theoretical model used for converting measured current into flux is described, followed by the details of experiments to calibrate a pin diode against a scintillator, measure diode thickness, assess the possible error in the simple model introduced by charge carrier recombination, and to check device linearity.

In the work reported here, eight diode types were used; the physical characteristics, manufacturers and model numbers of these are summarized in Table 1 ▶.

### 2.1. Theoretical model

The photon flux transmitted by the silicon layer of a pin diode, ϕ_trans_, is related to the cross section, *A*, the density of silicon^2^, ρ_Si_, and the diode thickness, *t*_Si_, by the following expression,
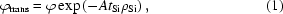
where ϕ is the incident photon flux. However, it is the energy deposited in the silicon layer rather than the transmitted intensity that generates current. Provided that the diode has a linear response (see §2.5 below), the ratio of incident photon flux to the total photocurrent will be a constant. It is convenient to express this constant in units of photons s^−1^ A^−1^ because multiplying this number by the observed diode current (in A) yields the flux (photons s^−1^). This quantity will be referred to as the photoconversion ratio (ratio of incident photon flux to diode current) of the diode.

**Figure 2:**
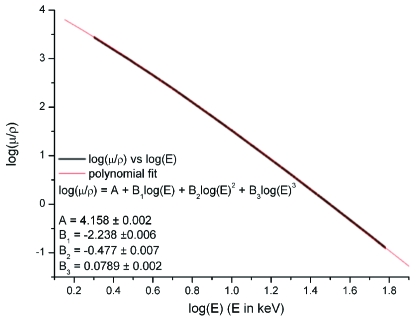
Log–log plot of the photoelectric cross section, μ_pe_/ρ_Si_ (units: cm^2^ g^−1^), of silicon as a function of incident X-ray energy.

Using equation (1) and the relationship *I* = ϕ*Q*, where *I* is the photo-induced current and *Q* is the charge created in an interaction for X-rays of energy *E* incident on a silicon diode, the photoconversion ratio can be expressed in the form
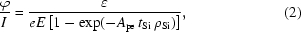
where *A*_pe_ is the photoelectric cross section of silicon, *e* is the electronic charge^3^ and ∊ is the energy required to generate an electron–hole pair defined in §1.5. The quantity *A*_pe_ is tabulated for all elements by the NIST *XCOM* program (http://physics.nist.gov/PhysRefData/Xcom/Text/XCOM.html) and is plotted for silicon as a function of energy in Fig. 2 ▶. Fitting a third-order polynomial in the log–log domain reproduces *XCOM* data to better than 1% in the range 5–40 keV and allows *A*_pe_ to be expressed in the form
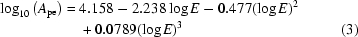
for silicon. Equations (2) and (3) therefore enable the beamline photon flux to be related to the current induced in the diode solely in terms of the beamline energy and the thickness of the diode.

The face of pin diodes is often covered with a protective aluminium layer to prevent ambient light adding to the signal recorded by the diode. When calculating the flux, the absorption owing to any such cover and also the extra loss owing to the air path between the diode and the sample must be taken into account. Thus flux attenuations owing to aluminium and air were calculated in an analogous way to that described by equation (1). The flux at the sample, ϕ_S_, is related to the flux, ϕ, measured at the diode position by the following expression,
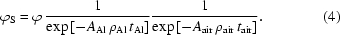
The X-ray cross sections (*A*_Al_ and *A*_air_) were obtained from the NIST photon cross-sections database. The calculation of the attenuation owing to air assumed a gas composition of 0.0124% carbon, 75.5268% nitrogen, 23.1781% oxygen and 1.2827% argon at a density of 1.205 × 10^−3^ g cm^−3^ (fractions by weight; composition as used by NIST). Attenuation in the diode p-layer has been assumed to be small and is neglected in the above parameterization.

### 2.2. Absolute calibration of the pin diode against a scintillator

The simple model described in §2.1 was validated through experiments carried out at beamline 8.3.1 of the ALS using a 0.1% thallium-doped NaI^4^ scintillator (Oxford Danfysik model CBY38NA01B) and an S100VL diode (see Table 1 ▶). The scintillator comprised a 1 mm-thick 30 mm-diameter crystal protected by a 0.2 mm-thick beryllium front window^5^. The effective window size of the scintillator was reduced by using a 5 mm round lead aperture so that the active area was identical to that of the S100VL diode. The photomultiplier tube (PMT) in contact with the NaI(Tl) crystal was connected to a single-channel analyzer (SCA) (Oxford Danfysik model CyberStar X1000). The SCA was set to have virtually no energy discrimination, with the ‘maximum’ peak height disabled and the ‘minimum’ peak height just above the noise level. A background of approximately 1 count s^−1^ was observed with these settings when the X-ray shutter was closed. The dead-time was determined empirically by exposing the detector to an increasing photon flux until the observed count rate reached a maximum. The experimentally determined dead-time ranged from 686 ns at 8 keV to 826 ns at 16 keV in an apparently linear fashion (data not shown).

To calibrate the diode, the scintillator was mounted in the direct beam path with the front window 37.4 mm downstream from the diode position, and the diode was mounted on an actuator to insert it into the beam as needed. Prior to the experiments described here, the profile of the uncollimated beam was determined by scanning a 10 µm tantalum pinhole across the sample position. A Gaussian profile with a FWHM of 120 × 108 µm (h × v) was obtained with a root-mean-square residual of 1.5% of the maximum flux through the pinhole (data not shown). In order to avoid significant scintillator photon pile-up, the beam was attenuated at each incident energy until ∼10^5^ counts s^−1^ were observed at the SCA. This ∼10^6^-fold attenuation was achieved by placing a 15 µm pinhole at the sample position, closing down the convergence defining slits, and detuning the rocking curve of the monochromator which also ensured that harmonic contamination was negligible. The absence of harmonic contamination was verified using an energy-resolving silicon drift diode detector (Evex Inc., Princeton, NJ, USA), and no counts were observed at the energy of the third harmonic (data not shown). The observed count rates were then divided by the expected fraction of photons detected given the dead-time. These rates were then divided by the fraction of photons expected to be absorbed by the scintillator crystal, further divided by the transmittance of the Be window and 37.4 mm of air, and then multiplied by the transmittance of the aluminium alloy^6^ foil covering the diode. The resulting count rate was taken as the incident photon flux on the front surface of the diode.

The current generated by the diode was measured with a low-noise current amplifier (Stanford Research Instruments model SR570) and was of the order of 25 pA. Accurately measuring such a small current required an amplifier input impedance of 1 MΩ, which formed a current divider with the diode. The small-signal (±1 mV) input impedance of this particular S100VL diode was measured separately and found to be 3.37 MΩ, which implies that 23% of the total photocurrent shunted through the diode itself and was never seen by the amplifier. For this reason the current reported by the amplifier was multiplied by 1.30 to obtain the total photocurrent generated by the diode^7^.

### 2.3. Diode thickness

There are several possible sources of error in using pin diodes to quantify photon flux. Foremost is the uncertainty in the diode thickness; if the diode thickness is different from the specification, then the calculated flux will not be the actual flux [equation (2)]. The thickness of both the X-ray sensitive layer and front window can be measured by tilting the diode in the X-ray beam by an angle θ, which effectively increases these thicknesses by a factor 1/cosθ, where θ is zero when the diode surface is normal to the X-ray beam,
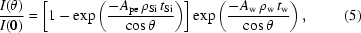
where the subscript w indicates the window material. This allows the thickness of both the silicon diode and the covering window to be determined experimentally.

### 2.4. Carrier recombination

Another potentially serious source of error is charge carrier recombination, and indeed a batch of ten S100VL diodes was found to be 30–50% less sensitive (data not shown) than the ‘good’ S100VL diode described here. Diodes that suffer from recombination have a much more complicated relationship between flux, current, thickness and photon energy (Cho *et al.*, 1992 ▶; Gullikson *et al.*, 1995 ▶; Lutz, 1999 ▶) than the photoconversion ratio described by equation (2). This equation can be used to identify them, since the thickness derived from tilt data by fitting equation (5) will be inconsistent with the energy dependence given by equation (2). To test this, a number of diodes with a range of thicknesses made by different manufacturers were compared at beamline X06SA of the SLS and beamlines 8.3.1 and 12.3.1 of the ALS. At beamline X06SA at the SLS, owing to spatial restrictions around the sample position, the diodes were placed 50 mm downstream directly behind the sample position, with the exception of the Sintef diode (Table 1 ▶) which was permanently mounted below the PILATUS 6M detector 145 mm downstream of the sample position for these experiments.

In all these experiments the relative sensitivity of diodes experiencing the same X-ray beam predicted by equation (2) was tested experimentally at photon energies between 4 and 18 keV.

### 2.5. Device linearity

**Figure 3:**
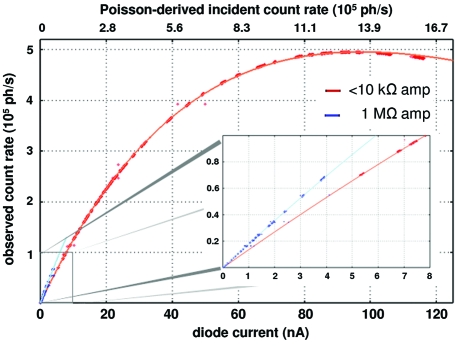
Representative plot of uncorrected scintillator counts per second *versus* diode current (points). A Poisson distribution (line) was fitted to these data and the overall agreement shown here implies that the diode current (lower *x*-axis) was linear with incident flux (proportional to upper *x*-axis). In this case the incident photon energy was 11 keV, a silica glass target was used as a differential attenuator as described in the text, and the ratio of incident photons diverted to the scintillator over the diode current was 13911 counts s^−1^ nA^−1^. The inset highlights a clear deviation from the overall best-fit Poisson model when the SR570 amplifier was using an input impedance of 1 MΩ (blue dots), indicating that the diode became non-linear in this region. This non-linearity was due to the current divider detailed in §2.2.

A final potential source of error is non-linearity. Current dividers between the amplifier and diode are a common source of non-linearity, especially at very high and very low currents, and care must be taken to understand the input impedance of the amplifier and the diode, and how the two interact (for an example see §2.2). Some diodes also internally saturate at a particular incident photon flux, so it was therefore important to establish the linearity of a diode by comparing it with a known linear device such as the dead-time-corrected scintillator. For this test the S100VL diode described above was used to monitor the X-ray beam transmitted through a piece of silica glass mounted at the sample position at ALS beamline 8.3.1, and the scintillator monitored the small fraction of photons scattered from the glass. This differential attenuator delivered a fixed fraction of incident photons to each of the two detectors for a given photon energy and experimental geometry, and the incident flux was varied by inserting or removing foils upstream or by adjusting an aperture 10 m upstream. Attenuations of the incident beam producing as low as ∼100 counts s^−1^ and up to saturation levels of the scintillator were explored, and a representative graph of diode current *versus* photon counts is shown in Fig. 3 ▶. These data were fitted to a Poisson distribution modified by a single free scale factor (13911 counts s^−1^ nA^−1^) to represent the ratio of photons incident on the two detectors in a given geometry. Data points deviating from this curve would indicate that either the diode was not linear or that the scintillator was deviating from simple Poissonian counting behaviour. The unlikely possibility of compensating non-linearities in the diode and scintillator was addressed by repeating this experiment with varying differential attenuation arrangements.

## 3. Results

To validate the proposed simple model for current to flux conversion, a series of experiments was required. First, the diode must be linear, as predicted by equation (2). Secondly, the effective thickness of the diode must be known. Thirdly, an absolute flux calibration must be performed and the results shown to be consistent with equation (2). Finally, the generality of the model must be validated by demonstrating that it gives the same flux for the same beam when using any one of a series of diodes of different thicknesses and from different manufacturers (Table 1 ▶).

### 3.1. Determination of diode linearity

The results of an experiment in which the photocurrent induced in a pin diode and the counts recorded by a scintillator were compared are shown in Fig. 3 ▶. The high-quality fit of the data to a Poisson distribution indicates that the scintillator obeys a Poissonian dead-time model and that the diode current is linear with flux over the range 5–120 nA. The discontinuity in the data at ∼5 nA corresponds to the change in the amplifier input impedance described in §2.2. Accounting for the current divider between the amplifier and the diode brought the 0.1–5 nA data exactly onto the Poissonian curve that best fit the rest of the data (not shown), which extended the demonstrated linearity of the total photocurrent of the diode down to ∼100 pA. Further diode–scintillator comparisons were made with different differential attenuator arrangements (data not shown) and the overlapping current ranges of these data demonstrated the linearity of the diode from 10 pA up to at least 0.1 mA.

Calibrating diodes using a counting device such as the scintillator described here requires that very small currents (pA) be measured accurately and the results extrapolated over six orders of magnitude to the full flux of the beamline, so this method of calibration is not a convenient general method for MX.

### 3.2. Measurement of diode thickness by tilting

**Figure 4:**
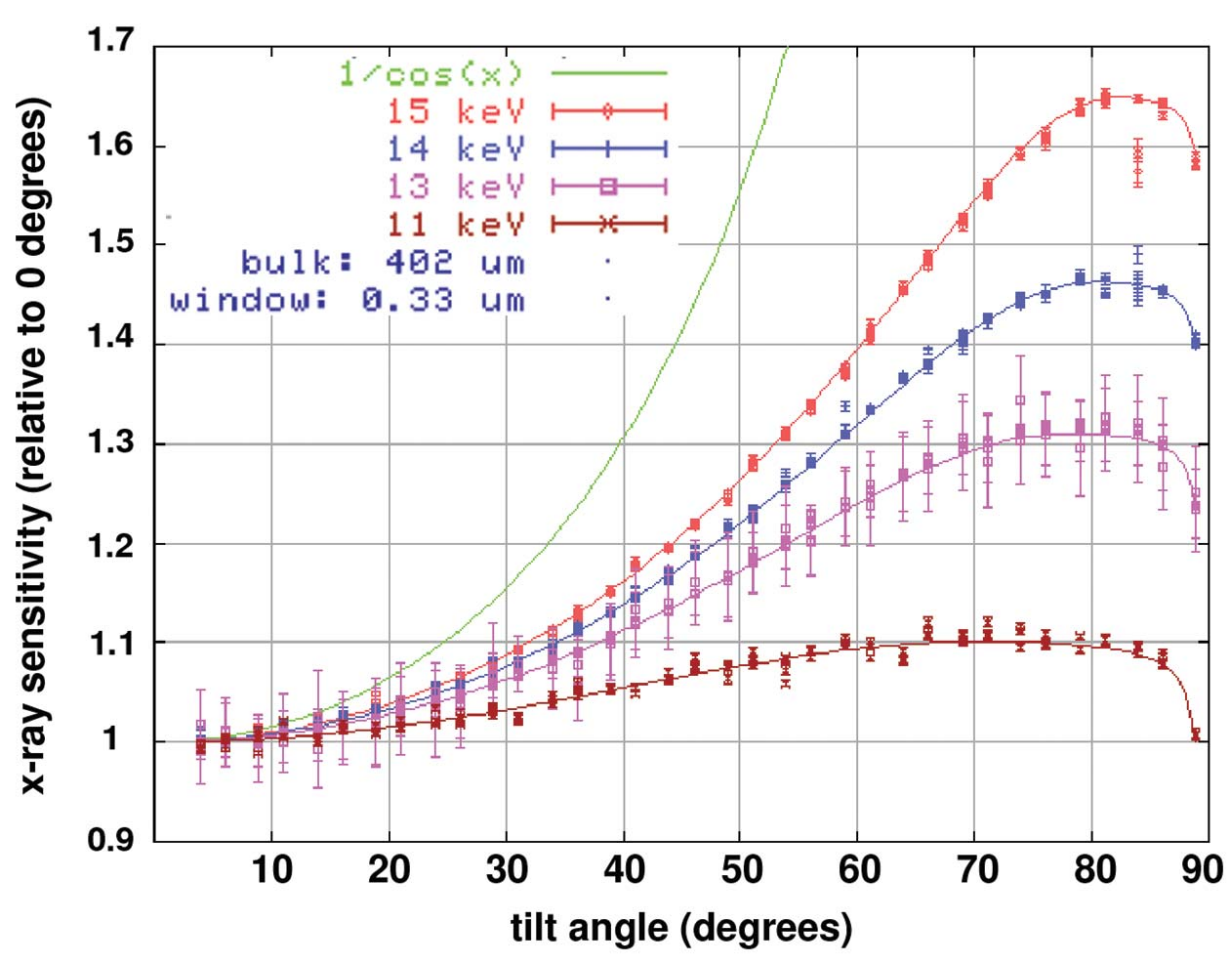
Normalized sensitivity of diode PIN-10DPI as a function of tilt angle at different incident X-ray energies. Line plots of equation (5) (*t*_Si_ 401.9 ± 0.5 µm, *t*_w_ 0.33 ± 0.01 µm) are overlaid on the experimental points for each energy.

Fig. 4 ▶ shows the relative sensitivity of an OSI PIN-10DPI diode (nominal active layer thickness, *t*_Si_, 400 µm, silicon ‘window’ thickness, *t*_w_, 0.33 µm) as a function of tilt angle. In this case no protective aluminium cover was used and the window here is a thin insensitive upper layer of silicon in the diode itself. It can be seen that the above values of *t*_Si_ and *t*_w_ are consistent with the tilt data at each photon energy. Note that in the extreme case when *t*_Si_ is negligibly small and the X-ray attenuation length [1/(*A*_pe_ρ_Si_)] is large, the beam intensity does not change appreciably as it moves through the diode and no information on *t*_Si_ is obtained. Conversely, if the attenuation length is very much smaller than *t*_Si_, then all the X-rays are absorbed, regardless of tilt, so *I*(θ)/*I*(0) = 1, which again gives no information on *t*_Si_.  However, if the attenuation length is comparable with *t*_Si_, then the *I*(θ)/*I*(0) curve falls between 1/cos(θ) and 1, allowing *t*_Si_ to be obtained from equation (5). In a similar way, maximum information regarding *t*_w_ is obtained at 11 keV because the slight peak at 70° is only consistent with equation (5) for *t*_w_ = 0.33 µm.

Tilt data were also collected for the OSI S100VL, IRD AXUV100 and Hamamatsu S3204-09 diodes and they were found to be consistent with 400, 300 and 300 µm sensitive layer thicknesses, respectively, as per the manufacturers’ specifications (data not shown). These thicknesses could then be used for validation of the simple energy-loss model for current-to-flux conversion.

### 3.3. Model validation

**Figure 5:**
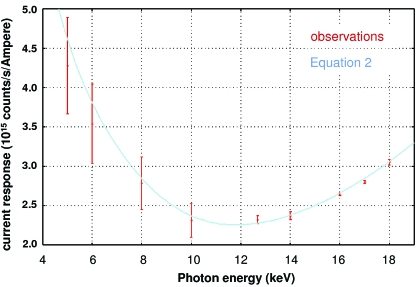
The ratio of the corrected scintillator count rate to current produced in the S100VL diode plotted against photon energy. Red error bars indicate the root-mean-square scatter of ten back-and-forth comparisons corrected for dead-time and window transmissions [equation (4)] while the blue line is the theoretical photoconversion ratio of the diode [equation (2)]. The theoretical photoconversion ratio is overlaid on the comparison, *not* fitted to the data. Note that the scatter in measured values is much greater at lower incident X-ray energies owing to the instability of the highly attenuated beam.

The proposed flux calculation model was applied to the S100VL diode characterized in §3.1. Fig. 5 ▶ shows the theoretical photoconversion ratio of the diode [equation (2)] in addition to the experimentally determined photoconversion ratio which was determined from comparison with the scintillator. The theoretical photoconversion ratio (blue line) is a superposition rather than a fit to the experimental data, illustrating the excellent agreement between the simple model and the experimental results.

**Figure 6:**
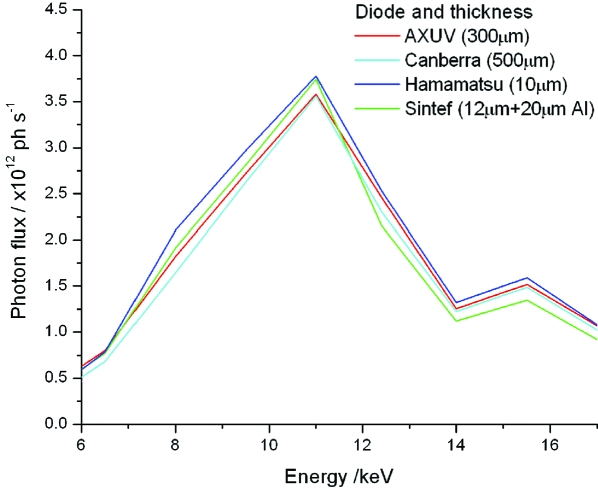
Photon flux calculated from equation (2) using the current observed from four diodes in the same X-ray beam as a function of X-ray energy. For details see Table 2 ▶. The energy dependence of the photon flux is a property of the undulator harmonics and the beamline optics and not of the pin diodes used.

Further evidence for the validity of the model is shown in Fig. 6 ▶, which displays the calculated fluxes from measurements taken at the SLS using four diodes of widely differing depths (10–500 µm), varying thicknesses of aluminium cover (0 to 23 µm) and for a range of collimator-to-diode distances, all as a function of incident X-ray energy. Since the photoconversion ratios [equation (2)] of these devices differed, but the flux of the X-ray beam was the same, the calculated flux rather than the photoconversion ratio is plotted. The results show very good agreement between all four diodes over the incident energy range scanned (5.8 to 17 keV).

Similar experiments were performed at the ALS by comparing one Hamamatsu S3204-09, fourteen OSI S100VL, one S4CL and ten PIN-10DPI diodes, taken in pairs. Apart from a batch of ten poor-quality S100VL diodes, the ratio of the currents generated by two diodes alternately placed in the same beam (4 to 18 keV) agreed with equation (2) to within 5% error (data not shown).

## 4. Discussion and conclusions

Dose is the recognized metric for quantifying rates of damage in MX. A prerequisite for estimation of the dose absorbed by a crystal is the incident photon flux. The results detailed above show that a simple model based on energy deposition in silicon is sufficient to accurately determine the X-ray flux incident on a high-quality pin diode from the electrical current induced within the diode.

Note that if significant charge-carrier recombination were taking place in the diodes detailed here, then the recovery of an electron–hole pair would depend on the depth at which it was created in the diode (Gullikson *et al.*, 1995 ▶), and equation (5) would not be valid. If recombination were taking place in the experiments described in §3.2, the best-fit values of *t*_Si_ and *t*_w_ would change with photon energy as would the shape of the tilt data. Both the close agreement of the data shown in Fig. 3 ▶ with equation (5), and the consistency of the calculated flux from different diodes with different thicknesses (Fig. 6 ▶) support the hypothesis that recombination can be neglected.

The simple model outlined above has thus been incorporated into a web-based calculator at http://x10sa.web.psi.ch/diode-calc.php. The calculator requires only the energy of the incident X-rays and the thickness of the diode to be known, though parameters such as the thickness of any protective aluminium layer covering the face of the diode and the distance of the diode from the sample position can also be factored into the calculation. The calculated flux can then be used to characterize a source during a particular experimental run, or to benchmark an internal beamline standard (for example, at the SLS, fluorescence from a thin film of chromium sputtered onto a kapton foil at 90° to the X-ray beam is used). Further miniaturization of pin diodes allows integration into the beamstop and routine measurement of flux during data collection (Ellis *et al.*, 2003 ▶).

In order to give users an idea of how the calculated flux relates to dose, the calculator relates flux to dose for a simple test case using a sample absorption coefficient calculated by *RADDOSE* (Murray *et al.*, 2004 ▶) for a 100 × 100 × 100 µm lysozyme crystal irradiated by a 100 × 100 µm X-ray beam with a top-hat profile. This calculation makes several assumptions, not least concerning the beam size and shape, but does provide a rough (correct within a factor of two for most samples not containing heavy atoms) indication of the time that a macromolecular crystal exposed to such a beam will last before absorbing the experimental dose limit of 30 MGy (reduction of initial diffraction intensity, *I*_0_ to 0.7*I*_0_), after which diffraction data will have questionable value (Owen *et al.*, 2006 ▶). For accurate dose determination, *RADDOSE* should be used with appropriate input values for the beam size, shape and profile, and crystal parameters (Paithankar *et al.*, 2009 ▶; Murray *et al.*, 2004 ▶).

The flux calculated using the above model is dependent on two user-input parameters: the thickness of the silicon layer and the induced current. Both of these are possible sources of error as, for example, the layer thickness of a diode may not be well characterized or the diode may have a low input impedance resulting in an underestimation of flux. In order to reduce uncertainty in flux measurements and allow comparison with a characterized diode, a number of calibrated PIN-10DPI are available for loan from JMH. A diode calibration service is also offered by some National Standards Laboratories.

The incorporation of the consideration of dose into programs providing crystallographic data collection strategies, such as *BEST* and *Web-ice* (Bourenkov & Popov, 2006 ▶; González *et al.*, 2008 ▶), means that a common flux scale is required at different light sources if a standard model of damage rates and crystal lifetimes in MX is to be reached. For this to be achieved, all MX beamlines should display the measured X-ray flux in the data acquisition GUI, and this value should be written to the image header.

The above characterization and comparison of several commercially available pin diodes, together with the provision of loan from a calibrated stock of diodes, makes possible the ready calibration of X-ray fluxes prior to experiment. In conjunction with a knowledge of the beam size and profile, and a suitable estimation of the absorption coefficient of samples, the dose absorbed by a crystal during the experiment can then be calculated.

## Figures and Tables

**Table 1:** Summary of the diodes used in this study The silicon layer thicknesses were provided by the manufacturer for all diodes except the OSI and IRD devices; the thicknesses of these were experimentally determined (see Fig. 4 ▶).

Diode	Manufacturer	Model number	Thickness of silicon layer (µm)	Thickness of aluminium cover (µm)
1	OSI Optoelectronics	S100VL (solderable chip series)	400	23.2
2	OSI Optoelectronics	PIN-10DPI	400	16.4
3	OSI Optoelectronics	S4CL	400	N/A
4	IRD	AXUV100	300	N/A
5	Sintef	CHICSi 12	12	20
6	Hamamatsu	S9724-010	10	N/A
7	Hamamatsu	S3204-09	300	N/A
8	Canberra	PD300-500CB	500	N/A

**Table 2:** Diode currents recorded as a function of energy at X06SA of the SLS, and the corresponding calculated photon fluxes

	AXUV diode		Sintef diode		Hamamatsu 10		Canberra 500	
Silicon thickness (µm)	300		12		10		500	
Aluminium cover (µm)	N/A		20		N/A		N/A	
mm of air	50		145		50		50	

Energy (keV)	Current (mA)	Flux (×10^12^ photons s^−1^)	Current (mA)	Flux (×10^12^ photons s^−1^)	Current (mA)	Flux (×10^12^ photons s^−1^)	Current (mA)	Flux (×10^12^ photons s^−1^)
5.8	0.123	0.560	0.0159	0.531	0.0353	0.514	0.0973	0.443
6.5	0.206	0.801	0.0272	0.767	0.0479	0.784	0.175	0.68
8	0.602	1.826	0.0716	1.918	0.0975	2.111	0.549	1.647
9.5	1.032	2.726	0.0932	2.816	0.104	2.974	1.055	2.63
11	1.409	3.584	0.1039	3.741	0.102	3.78	1.602	3.56
12.4	0.938	2.460	0.0502	2.157	0.0547	2.532	1.083	2.306
14	0.44	1.254	0.0214	1.123	0.0226	1.321	0.568	1.224
15.5	0.48	1.520	0.0215	1.351	0.0223	1.592	0.653	1.485
17	0.30	1.068	0.0123	0.918	0.0126	1.081	0.416	1.023

## Footnotes

1 10^12^ photons s^−1^ × 12398 eV photon^−1^ × 1.6 × 10^−19^ J eV^−1^ = 2 mW.

2 Density: 2.33 g cm^−3^; photoelectric cross section: 16.5 cm^2^ g^−1^ at 12.66 keV.

3
               *e* = 1.6022 × 10^−19^ C.

4 Density of crystalline NaI: 3.67 g cm^−3^; photoelectric cross section: 72.6 cm^2^ g^−1^ at 12.66 keV.

5 Density: 1.86 g cm^−3^; total cross section 0.4 cm^2^ g^−1^ at 12.66 keV.

6 Thickness: 23.24 µm; density: 2.7 g cm^−3^; 98.5% Al, 0.75% Fe, 0.75% Si; total cross section: 13.7 cm^2^ g^−1^ at 12.66 keV.

7 Fraction of current observed by amplifier: 1/[(1/3.37) + 1] = 1/1.30 = 0.77.
